# Study to Evaluate Molecular Mechanics behind Synergistic Chemo-Preventive Effects of Curcumin and Resveratrol during Lung Carcinogenesis

**DOI:** 10.1371/journal.pone.0093820

**Published:** 2014-04-04

**Authors:** Anshoo Malhotra, Praveen Nair, Devinder Kumar Dhawan

**Affiliations:** 1 Department of Biophysics, Post Graduate Institute of Medical Education and Research [PGIMER], Chandigarh, India; 2 Department of Biophysics, Panjab University, Chandigarh, India; Indian Institute of Toxicology Research, India

## Abstract

**Background:**

The combination approach is the future of the war against cancer and the present study evaluated molecular mechanics behind the synergistic effects of curcumin and resveratrol during lung carcinogenesis.

**Methods:**

The mice were segregated into five groups which included normal control, Benzo[a]pyrene[BP] treated, BP+curcumin treated, BP+resveratrol treated and BP+curcumin+resveratrol treated.

**Results:**

The morphological analyses of tumor nodules confirmed lung carcinogenesis in mice after 22 weeks of single intra-peritoneal[i.p] injection of BP at a dose of 100 mg/Kg body weight. The BP treatment resulted in a significant increase in the protein expressions of p53 in the BP treated mice. Also, a significant increase in the protein expression of phosphorylated p53[ser15] confirmed p53 hyper-phosphorylation in BP treated mice. On the other hand, enzyme activities of caspase 3 and caspase 9 were noticed to be significantly decreased following BP treatment. Further, radiorespirometric studies showed a significant increase in the ^14^C-glucose turnover as well as ^14^C-gulcose uptake in the lung slices of BP treated mice. Moreover, a significant rise in the cell proliferation was confirmed indirectly by enhanced uptake of ^3^H-thymidine in the lung slices of BP treated mice. Interestingly, combined treatment of curcumin and resveratrol to BP treated animals resulted in a significant decrease in p53 hyper-phosphorylation, ^14^C glucose uptakes/turnover and ^3^H-thymidine uptake in the BP treated mice. However, the enzyme activities of caspase 3 and caspase 9 showed a significant increase upon treatment with curcumin and resveratrol.

**Conclusion:**

The study, therefore, concludes that molecular mechanics behind chemo-preventive synergism involved modulation of p53 hyper-phosphorylation, regulation of caspases and cellular metabolism enzymes.

## Introduction

Cancer is the second amongst various fatal diseases in the world. At present, more than 10 million people are diagnosed with cancer every year [1]. Lung cancer is by far the leading cancers causing death in both men and women [2]. Cancer chemoprevention is an emerging approach to contain this deadly disease which involves adequate intake of dietary constituents including phytochemicals to reverse/inhibit the carcinogenic process [3], [4]. Phytochemicals are non nutritive products of plants and are presently being studied the world over for their chemopreventive actions in controlling various diseases including cancer. Amongst various chemo-preventive agents, phytochemicals especially curcumin and resveratrol in combination have shown great potential in combating cancer as observed in our previous studies [5]–[9]. However, there is paucity of information with regard to molecular mechanics behind these synergistic effects of curcumin and resveratrol. So, the present study is aimed to study possible mechanisms behind chemo-preventive synergism of curcumin and resveratrol.

Tumor suppressor proteins are the key targets of various chemo-preventive agents and most of these proteins are regulated by post translational modifications [10]. The tumor suppressor protein p53 contains distinct sites where post translational modifications have been reported [11]. The study of these modifications at specific sties can provide mechanistic information about synergistic effects of curcumin and resveratrol. Therefore, the prime aim of the present study is to investigate phosphorylation at serine 15 residue of tumor suppressor p53 which is an important post translation modification responsible for functional efficacy and stability of p53. Further, the study of apoptosis response is essential to confirm efficient stimulation of p53 that was confirmed by evaluation of activities of caspases. Secondly, cancer cells have high turnover due to rapid proliferation and altered cellular metabolism. The cellular metabolism of cancer cells was studied biophysically by radiorespirometric analyses and in-vitro ^14^C labeled glucose uptake. The rapid proliferation of cancer cells was measured by recording in-vitro uptake of ^3^H thymidine by lung tissues, which was further confirmed biochemically by evaluation of enzyme activity of lactate dehydrogenase.

So, the present study is first of its kind to unravel the molecular mechanics responsible for chemo-preventive synergism of curcumin and resveratrol during lung carcinogenesis.

## Materials and Methods

### Chemicals

Benzo[a]pyrene, curcumin and resveratrol were procured from Sigma Aldrich company. Antibodies p53, phosphorylated[ser-15]-p53 and colorimetric kits for caspase 3 as well as Caspase 9 were procured from Biovision [USA]. ^3^H labelled thymidine and ^14^C labeled Glucose were procured from Board of Radiation Isotope Technology [BRIT], Trombay, India. All other reagents were procured from Merck Chemicals and Loba chemicals Pvt. Ltd.

### Animals

Male laka mice in the weight range of 18–20 g were procured from the central animal house, Panjab University, Chandigarh, India. The animals were housed in polypropylene cages under hygienic conditions in the departmental animal house. The study was approved by Institutional Animal Ethics committee [IAEC], Panjab University, Sector 14, Chandigarh.

### Experimental design

Animals were segregated equally [n = 10] and randomly into five treatment groups. Animals in Group I served as normal controls and were also administered intraperitoneally corn oil [I.P], which was used as a vehicle for the Benzo[a]pyrene treated animals. Animals in Group II were given a single intraperitoneal injection of Benzo[a]pyrene in corn oil at a dose level of 100 mg/Kg body weight [12]. Group III animals were given curcumin orally in drinking water at a dose level of 60 mg/Kg/body weight [13], thrice a week. Animals in Group IV were given resveratrol orally at a dose level of 5.7 μg/ml drinking water [14], thrice a week. Both the phytochemicals were given to animals using intubation gavage technique. Animals in group V animals were given a combined treatment of curcumin and resveratrol in a similar manner as was given to group III and group IV animals, respectively. The animals were subjected to treatment with phytochemicals, ten days prior to single benzo[a]pyrene injection. All the animals had free access to the diet and water and the treatments with phytochemicals were continued for a total duration of 22 weeks.

### Lung tumor burden analyses

After the terminal sacrifice following 22weeks, lungs were excised from the mice, blotted dry, and were examined for the visible macroscopic lesions. The number of tumors was noted for tumor burden studies.

### Preparation of nuclear extracts

20% lung [lung nodules in case of animal with tumors] homogenates were prepared in ice cold buffer A [10 mM HEPES, 50 mM NaCl, 500 mM sucrose, 1 mM EDTA, 0.2% tritone X][pH 8] using Teflon fitted potter-Elvejhem type homogenizer for few minutes till total disruption of cells. Homogenates were centrifuged at 5000 rpm for 2 minutes at 4°C. Pellets were resuspended in 500 uL of buffer B[50 mM NaCl, 10 mM HEPES, 0.1 mM EDTA, 25% glycerol][pH 8] followed by centrifugation at 5000 rpm for 3 minutes at 4°C. Pellets were again resuspended in 50 uL of buffer C [350 mM NaCl, 0.1 mM EDTA,10 mM HEPES, 25% glycerol][pH 8], containing 10 uL of protease inhibitor. The above suspensions were incubated on ice for 30 minutes with constant shaking, followed by centrifugation at 1000 rpm for 3 minutes at 4°C. Pellets were discarded and the supernatants were used for protein expressions studies of nuclear proteins.

### Western Transfer analyses

The protein expressions of anti P53[1∶500], anti phosphor-P53[ser-15] [1∶500] and anti-mouse-beta-actin[1∶10,000] were checked in the nuclear extracts after 22 weeks of all treatments. Protein concentrations in these fractions were determined by the method of Lowry et al [15] and were subjected to electrophoresis separation on SDS-PAGE followed by electro-transfer to PDVF membranes. The densitometric analyses of the bands were analyzed using Image J software.

### Immuno-histochemical analyses

Localized protein expressions of P53 as well as phosphorylated -P53[ser-15] were checked in the 7 micrometer thick paraffin sections of mice lungs using P53[1∶500], anti phosphor-P53[ser-15] [1∶500]. IHC slides were analyzed quantitatively by using single channel color analyses of adobe photoshop 7.

### Caspase 3 and Caspase 9 enzyme assay

Enzyme activities of Caspase-3 as well as Caspase 9 were assayed by using ‘Biovision Colorimetric Assay Kit ’ which provided a simple and convenient means for assaying the activity of caspases.

### In-vitro uptake of ^14^C-D-Glucose by the method of Crane and Mandelstam [16]

The lungs of normal as well as treated mice were firstly removed and then sliced into very thin sections and the uptake of ^14^C-D-Glucose was measured in the weighed slices by tissue accumulation method. Briefly, 8 μCi of radio-labeled glucose and 5 mM of unlabelled D-glucose were added into 100 ml of stock Kreb's ringer buffer. Tissue slices were then allowed to incubate for 10 min at 37°C in stoppered flasks containing 5.0 ml of oxygenated [95% O_2_ and 5% CO_2_] Kreb's ringer bicarbonate buffer [pH 7.4] having cold glucose and trace amounts of radio-labeled glucose/[0.4 μCi/5 ml in flask]. Lung slices were then taken out, filter dried and were dissolved overnight in different scintillation vials containing 20% KOH. Bray's scintillation fluid [30 g Napthalene, 200 mg POPOP, 2 g PPO, 10 ml ethylene glycol, 10 ml glacial acetic acid and 50 ml methanol and final volume adjusted to 500 ml with 1,4 Dioxan] was then added to each vial and the radioactivity was measured using liquid scintillation counter. Prior to counting, the liquid scintillation counter was set for ^14^C energy and the background counts of empty vials were recorded.

### Radiorespirometry of ^14^C-D-Glucose metabolism

Radiorespirometric method is the most sensitive method of analytical procedures. The application of this method to the in vitro measurement of metabolism can be accomplished by supplying ^14^C-labeled substrates and collecting and measuring the radioactivity of the evolved ^14^CO_2_. In this technique, a small vial was placed inside a standard liquid scintillation vial to form two compartments. ^14^C-labeled substrate and the tissue sample were placed in one compartment, the inner vial and the other compartment, the outer vial contained a Whatman-I filter wick impregnated with scintillation flours and alkali. ^14^CO_2_ produced through respiration in the sample compartment is trapped on the wick and was measured in a liquid scintillation counter.

### Uptake of ^3^H-thymidine to assess cell proliferation by the method of Crane and Mandelstam

The cell proliferation was assessed in-vitro by measuring the incorporation of ^3^H-thymidine in the lungs of the normal control and treated animals. The lungs were sliced into very thin sections and the uptake of ^3^H-Thymidine was measured in the weighed slices by tissue accumulation method [16]. Briefly, 0.74 MBq of radiolabeled thymidine was added into 100 ml of stock Kreb's ringer buffer. Lung slices were then allowed to incubate for 10 min at 37°C in stoppered flasks containing 5.0 ml of oxygenated [95% O_2_ and 5% CO_2_] Kreb's ringer bicarbonate buffer [pH 7.4] having trace amounts of radio-labeled thymidine [0.37 MBq/5 ml in flask]. Lung slices were then filter dried and were dissolved overnight in different scintillation vials containing 20% KOH. Bray's scintillation fluid was then added in each vial and the radioactivity was measured using liquid scintillation counter. Prior to counting, the liquid scintillation counter was set for ^3^H energy and background counts of empty vials were recorded.

### Alkaline phosphatase [ALP] and Lacatate Dehydrogenase [LDH]

Alkaline phosphatase activity was assayed according to the method of Bergmeyer [17]. LDH activity was measured by tracing the rate of oxidation of NADH at 340 nm as described by Bergmeyer & Bernt [18].

### Statistical analysis

The statistical significance of the data has been determined using one-way analysis of variance [ANOVA] followed a multiple post-hoc least significant difference test. The results are represented as Means ± S.D.

## Results

**Figure 1:**
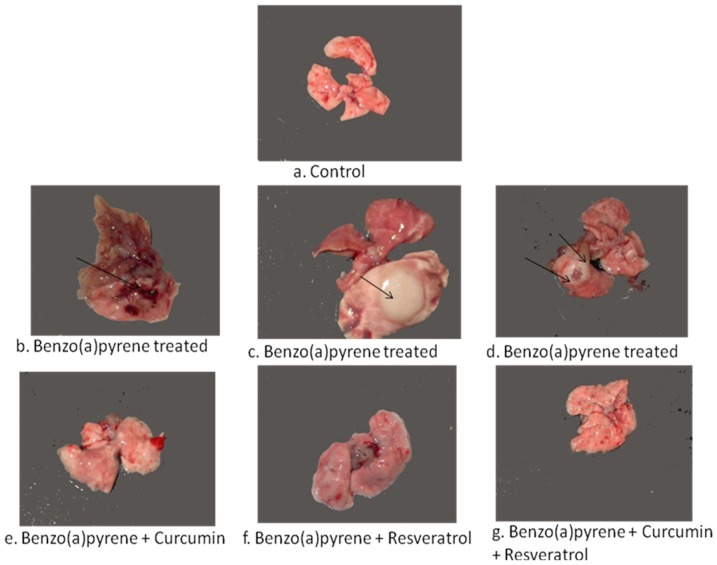
Macroscopic view of lungs. [a] normal lung [b] Benzo[a]pyrene treated showing angeogenic lesions and marked inflammation [c] Benzo[a]pyrene treated lung showing tumors [close up] [d] Benzo[a]pyrene treated lung showing tumor, [e] Bezo[a]pyrene + Curcumin [f] Benzo[a]pyrene + Resveratrol [g] Benzo[a]pyrene +Curcumin + Resveratrol.

**Figure 2:**
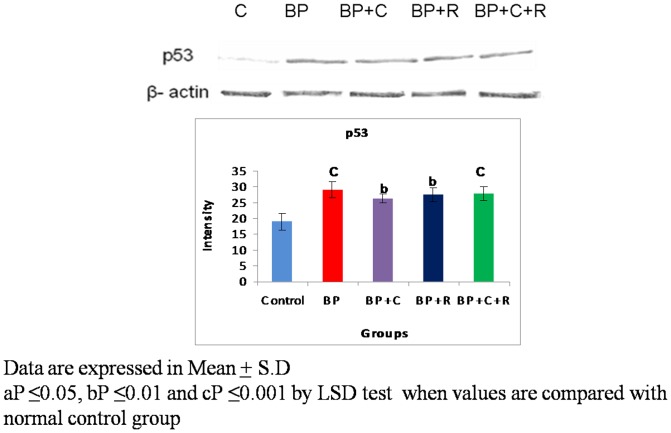
Effects of curcumin and resveratrol on protein expression of p53 by western blot analysis during lung carcinogenesis.

**Figure 3:**
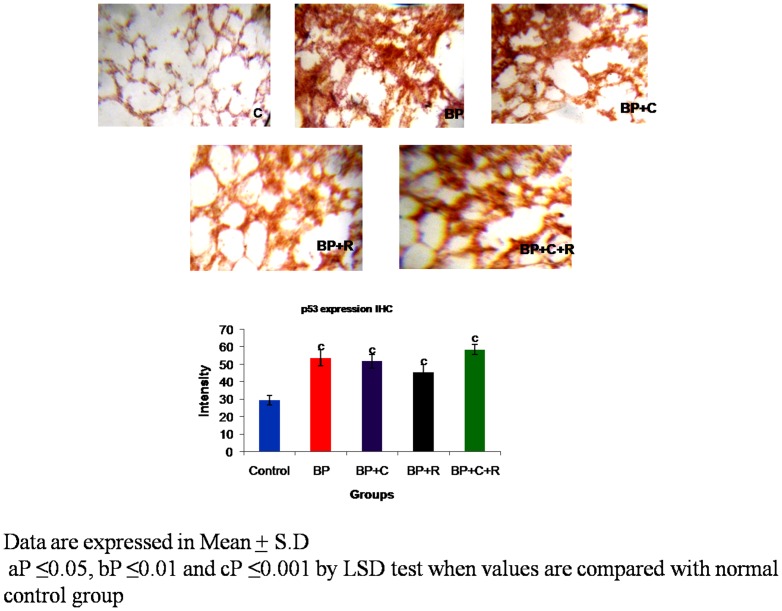
Effects of curcumin and resveratrol on protein expression of p53 by Immunhistochemistry [IHC at 10× magnification] during lung carcinogenesis.

**Figure 4:**
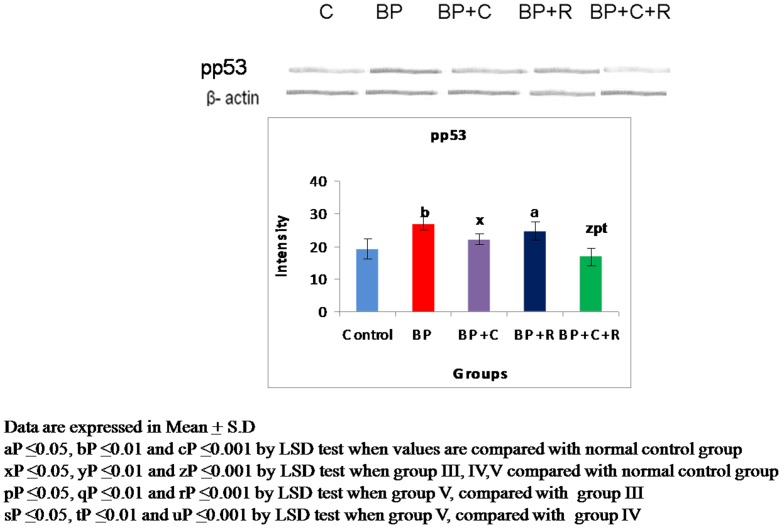
Effects of curcumin and resveratrol on protein expression of phoshorylated p53[ser-15] by western blot analysis during lung carcinogenesis.

**Figure 5:**
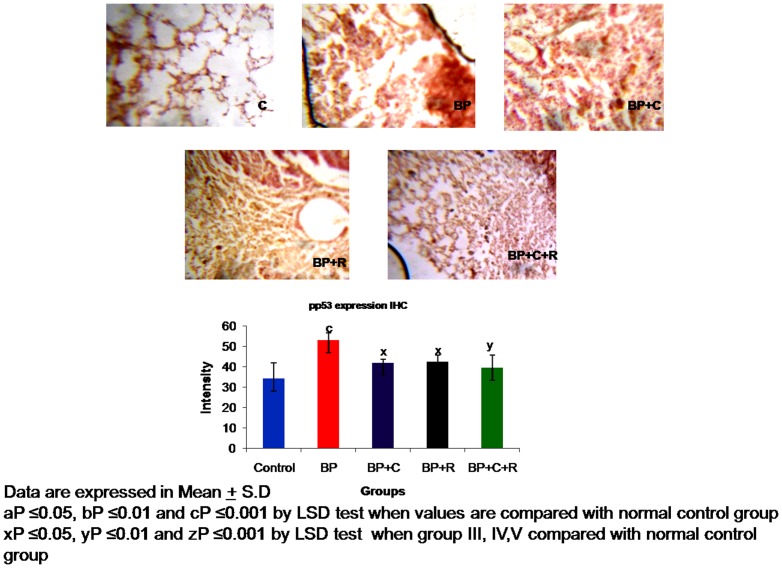
Effects of curcumin and resveratrol on protein expression of phoshorylated p53[ser-15] by Immunhistochemistry [IHC at 10 × magnification] during lung carcinogenesis.

The results obtained from various experiments conducted in this study are depicted in Tables 1, 2, 3, 4, 5 and figures 1, 2, 3, 4, 5. The data from various treatment groups have been compared with the normal control animals. Further, results obtained from BP+curcumin, BP+resveratrol and BP+curcumin+resveratrol treated groups were compared with that of BP group and the results of BP+curcumin and BP+ resveratrol treated groups were also compared with that of BP+ curcumin+ resveratrol combined treatment group.

**Table 1:** Effects of curcumin and resveratrol treatment for 22 weeks on the tumor incidence and multiplicity in mice subjected to benzo[a]pyrene treatment with single dose.

Groups	Tumor Burden [Total no. of tumors in each group]
**Normal control**	**0**
**Benzo[a]pyrene**	**14**
**Benzo[a]pyrene + Curcumin**	**10**
**Benzo[a]pyrene + Resveratrol**	**8**
**Benzo[a]pyrene + Curcumin + Resveratrol**	**7**

**Table 2:** Effects of curcumin and resveratrol treatments for 22 weeks on the enzyme activities of caspase 3 and caspase 9 in lungs during benzo[a]pyrene induced lung carcinogenesis.

Groups	Caspase 3 [n moles of pNA formed/min/mg protein]	Caspase 9 [n moles of pNA formed/min/mg protein]
**Normal control**	5.33±0.29	2.06±0.10
**Benzo[a]pyrene**	4.17±0.29^b^	1.42±0.10^c^
**Benzo[a]pyrene + Curcumin**	5.38±0.10^y^	1.78±0.09^b^ ^,y^
**Benzo[a]pyrene + resveratol**	4.42±0.44^b^	1.77±0.08^b^ ^,z^
**Benzo[a]pyrene + Curcumin + resveratrol**	5.06±0.63^x^	1.73±0.15^c,z^

n = 10 Data are expressed in Mean ± S.D.

b P<0.01 and ^c^P<0.001 by Least Significance Difference test when values are compared with normal control group.

x P<0.05, ^y^P<0.01, ^z^P<0.001 by Least Significance Difference test when values of Groups III, IV & V are compared with Group II.

**Table 3:** Effects of curcumin and resveratrol treatments for 22 weeks on the uptake of ^14^C-glucose in lungs during benzo[a]pyrene induced lung carcinogenesis.

Groups	^14^CO_2_ [% age of ^14^CO_2_ trapped as Na_2_^14^CO_3_/min/g tissue]	^14^C-glucose [μ moles of glucose incorporated/min/g tissue]
**Normal control**	1.19±0.07	0.20±0.02
**Benzo[a]pyrene**	2.01±0.28^b^	0.39±0.05^c^
**Benzo[a]pyrene + Curcumin**	1.59±0.57	0.33±0.05^b^
**Benzo[a]pyrene + Resveratrol**	1.42±0.40^x^	0.28±0.01^x^
**Benzo[a]pyrene + Curcumin +Resveratrol**	1.33±0.11^x^	0.28±0.0^a^ ^,y^

n = 10, Data are expressed in Mean ± S.D.

a P<0.05, ^b^P<0.01 and ^c^P<0.001 by Least Significance Difference test when values are compared with normal control group.

x P<0.05, ^y^P<0.01 by Least Significance Difference test when values of Groups III, IV & V are compared with Group II.

**Table 4:** Effects of curcumin and resveratrol treatments on the *in vitro* ^3^H-thymidine uptake in lungs during benzo[a]pyrene induced lung carcinogenesis.

Groups	[% age specific activity]
**Normal control**	3.03±0.31
**Benzo[a]pyrene**	3.94±0.23^b^
**Benzo[a]pyrene + Curcumin**	3.73±0.48^a^
**Benzo[a]pyrene + Resveratrol**	3.90±0.27^c^
**Benzo[a]pyrene + Curcumin + Resveratrol**	3.52±0.30^a^

n = 10, Data are expressed in Mean ± S.D.

a P<0.05, ^b^P<0.01 and ^c^P<0.001 by Least Significance Difference test when values are compared with normal control group.

**Table 5:** Effect of 22 weeks of curcumin and resveratrol treatments on the activity of alkaline phosphatase [ALP], lactate dehydrogenase[LDH] in serum of mice subjected to benzo[a]pyrene treatment.

Groups	ALP [μmoles of p-nitrophenol produced/min/mg protein]	LDH [μ moles of NADH oxidized/min/mg protein]
**Normal control**	0.92±0.08	2.99±0.23
**Benzo[a]pyrene**	1.47±0.33^b^	5.35±0.48^c^
**Benzo[a]pyrene + Curcumin**	1.06±0.24^x^	2.45±0.51^z^
**Benzo[a]pyrene + Resveratrol**	1.27±0.12^a^	2.71±0.72^z^
**Benzo[a]pyrene + Curcumin + Resveratrol**	1.08±0.27^x^	3.30±0.49^z,^ p

n = 10,Data are expressed in Mean ± S.D.

a P≤0.05, ^b^P≤0.01 and ^c^P≤0.001 by Least Significance Difference test when values are compared with normal control group.

x P≤0.05, ^z^P≤0.001 by Least Significance Difference test when values of Groups III, IV & V are compared with Group II.

p P≤0.05 by Least Significance Difference test when values of Groups V is compared with Group III.

### Macroscopic view of tumors

Figure 1[a] shows a lung from normal mice. Further, figures 1[b], 1[c] and 1[d] depict developed tumor nodules in lungs alongwith angiogenic lesions and the spread of inflammation on the lung surface in the mice treated with benzo[a]pyrene. Figures 1[e]–1[f], show appreciable improvement in the lungs with a decrease in the number of tumor nodules and angiogenic lesions in the benzo[a]pyrene exposed mice given treatment with phytochemicals separately [figure 1[e], 1[f]] and in combination figure 1[g].

### Lung tumor Burden analyses

The benzo[a]pyrene treatment resulted in tumor burden of 14 tumors [Table 1]. Supplementation of BP treated mice with curcumin and resveratrol individually showed slight decrease in the tumor burdens which were found to be 10 and 8 respectively. Further, decrease in the tumor burden was observed when BP treated mice were subjected to combined treatment of Phytochemicals that was observed to be 7.

### Western Transfer analyses and immuno-histochemical studies

The benzo[a]pyrene treatment resulted in a significant increase in the protein expressions of p53 [Figures 2–3] as well as phosphorylated-p53 [Figure 4–5] as observed by western transfer and IHC in lung tissue. Further, supplementation with phytochemicals to benzo[a]pyrene treated animals showed no significant change in the expression of p53 but a statistically significant decrease in the protein expression of phosphorylated p53 was noticed.

### Apoptotic Marker Enzymes [Caspase 3 and Caspase 9]

A significant decline in the enzyme activities of both caspase 3 and caspase 9 was observed [Table 2] in the lungs of mice treated with benzo[a]pyrene. Supplementation with phytochemicals brought a significant improvement in the enzyme activities of both caspase 3 and caspase 9.

### Radiorespirometric study of ^14^C glucose turnover and in vitro Uptake of ^14^C glucose

Benzo[a]pyrene treatment resulted in a significant increase [Table 3]in both the ^14^C glucose turnover as well as ^14^C glucose uptake. On the other hand combined treatment of curcumin and resveratrol resulted in a significant decrease in both uptake and turnover values of ^14^C-glusose as compared to benzo[a]pyrene treated group.

### Uptake of ^3^H-Thymidine to assess cell proliferation

A statistically significant increase [Table 4] in the uptake of ^3^H-thymidine was observed in the lungs of benzo[a]pyrene treated mice. However, supplementation with phytochemicals did cause some decrease in the ^3^H-thymidine uptake values but the decrease was not statistically significant.

### Alkaline phosphatase[ALP] and Lactate Dehydrogenase [LDH] enzyme activities

A statistically significant increase in the enzyme activity of ALP as well as LDH was observed in benzo[a]pyrene treated mice [Table 5]. Combined supplementation of curcumin and resveratrol resulted in statistically significant moderation of enzyme activity of alkaline phosphate in benzo[a]pyrene treated mice.

## Discussion

Combination approach is an emerging area of cancer research which is currently being explored the world over. It includes combined use two molecules [drugs/Phytochemicals/chemo-preventive agents] to enhance efficacy of the treatment. Therefore, in the present study, experimental model of lung carcinogenesis was developed to explore the molecular mechanics responsible for chemo-preventive synergistic effects of curcumin and resveratrol. The study clearly indicated that the administration of curcumin and resveratrol in combination to the BP treated animals appreciably modulated p53 hyperphosphorylation, thereby, regulated apoptosis as evidenced by improvement in the enzyme activities of apoptotic enzymes viz. caspases 3 and 9. All these molecular events collectively contributed towards chemoprevention synergism against lung cancer in mice.

The p53 tumour suppressor is a tightly regulated protein that acts by inhibiting cell-cycle progression or by promoting apoptosis whenever cells encounter DNA damage, oncogene activation or during carcinogenesis. The importance of p53 in cancer development is illustrated by the fact that p53 is highly mutated [≥18,000 mutations] in various cancers including lung cancer. It is probably rendered inactive by a range of indirect mechanisms including post translational modifications [19]. One of the major post-translational modifications being studied by many researchers is phosphorylation of serines of p53. The present study observed a significant increase in the protein expression of p53 as well as of phosphorylated p53 indicative of p53 hyper-phosphorylation at serine-15 residue in lungs of BP treated mice. The observed increase in p53 expression is a natural defence to prevent uncontrolled proliferation of transformed cells [20]. However, the above increase of p53 expression is ineffective due to presence of inactive [genetically or post-translationally modified] forms of p53. Further, the observed p53 hyper-phosphorylation at ser-15 residue could be one of the major contributors for p53 inactivation as there are studies which reported that hyper-phosphorylation at ser-15 results in p53 inactivation [21], [22]. Hyper-phosporylation at serine 15 residue of p53 influences the interactions of trans-activation domain of p53, thereby, rendering it inactive. On the basis of above reports the present study inferred that hyper-phosphorylation might be interfering with the tumour suppressor function of p53. Supplementation with curcumin and resveratrol in combination brought a significant moderation in the p53 hyper-phosphorylation in lungs of benzo[a]pyrene treated mice. This decrease in the p53 hyper-phosphorylation might be one of the prime molecular events utilized by both curcumin and resveratrol to enhance the efficacy of tumour suppressor p53 against lung carcinogenesis.

Caspase 3 and Caspase 9 are marker apoptosis enzymes of both intrinsic as well as extrinsic pathway respectively and their activities have been found to be significantly decreased in the BP treated mice. However, curcumin and resveratrol synergistically stimulated enzyme activities of both caspase 3 and caspase 9. The regulation of p53 phosphorylation by combined treatment of curcumin and resveratrol resulted in activation of p53 that caused induction of apoptosis enzymes. The study by Sen et al[23] also observed stimulation of caspases by phytochemicals treatment in lung cancer cells. The observation is in corroboration with our earlier reports which noticed trigger of apoptosis by stimulation of Bax gene and inhibition of Bcl_2_ gene by curcumin and resveratrol during lung carcinogenesis [6]–[9]. So, induction of apoptosis via both intrinsic as well as extrinsic pathways could be the second molecular event behind chemo-prevention synergism of curcumin and resveratrol.

Radiorespirometric and uptake studies of ^14^C-glusocse showed a significant increase in the turnover and uptake of ^14^C-glusose, respectively, in lung slices of benzo[a]pyrene treated mice. The above increase is the indicator of enhanced requirement of glucose by rapidly proliferating cancer cells. The root cause for the increase in glucose demand is again p53 hyper-phosphorylation that resulted in decline in apoptosis and hence, ended in rapid proliferation of cells. This rapid cell proliferation was further confirmed biophysically by a significant increase in the uptake of ^3^H thymidine in the lung slices of benzo[a]pyrene treated mice. Moreover, cell proliferation was also observed biochemically by elevated enzyme activity of lactate dehydrogenase [enzyme is marker of tissue turnover] in the BP treated mice. Combined treatment of curcumin and resveratrol resulted in appreciable moderation in the uptake as well as turnover of glucose in the lungs of benzo[a]pyrene treated mice. This can be owed to synergistic effects of curcumin and resveratrol which involved induction of apoptotic control via regulation of p53 phosphorylation over rapidly proliferating cells. This inference was further supported by a significant decrease in the ^3^H-thymidine uptake as well as enzyme activity of LDH in combined phytochemicals supplemented benzo[a]pyrene group. The results are in sync with earlier reports [24]–[27].

Deregulation of p53 phosphorylation also countered biochemically in BP treated mice by a significant increase in the enzyme activity of alkaline phosphatase[ALP]. It is a hydrolase enzyme responsible for dephosphorylation of various bio-molecules, including nucleotides, proteins etc. So, cell is trying to counter balance the excess of p53 phosphorylation by stimulating alkaline phosphatase. However, supplementation with phytochemicals resulted in a significant decrease in its activity which could be linked to ability of curcumin and resveratrol to regulate p53 phoshphorylation.

The present study added another dimension to the existing knowledge of phytochemicals in cancer chemoprevention. The study concludes that phytochemicals in combination regulate p53 phoshphorylation specifically at ser 15 to control apoptosis in rapidly proliferating cancer cells during lung carcinogenesis.
